# Evidence for Direct Control of Virulence and Defense Gene Circuits by the *Pseudomonas aeruginosa* Quorum Sensing Regulator, MvfR

**DOI:** 10.1038/srep34083

**Published:** 2016-09-28

**Authors:** Damien Maura, Ronen Hazan, Tomoe Kitao, Alicia E. Ballok, Laurence G. Rahme

**Affiliations:** 1 Department of Surgery, Massachusetts General Hospital, Boston MA 02114, USA; 2 Department of Microbiology and Immunobiology, Harvard Medical School, Boston MA 02115, USA; 3 Shriners Hospitals for Children Boston, Boston, 02114, Massachusetts, USA; 4 Institute of Dental Sciences and School of Dental Medicine, Hebrew University, Jerusalem P.O.B 12272, 91120, Israel

## Abstract

*Pseudomonas aeruginosa* defies eradication by antibiotics and is responsible for acute and chronic human infections due to a wide variety of virulence factors. Currently, it is believed that MvfR (PqsR) controls the expression of many of these factors indirectly via the *pqs* and *phnAB* operons. Here we provide strong evidence that MvfR may also bind and directly regulate the expression of additional 35 loci across the *P. aeruginosa* genome, including major regulators and virulence factors, such as the quorum sensing (QS) regulators *lasR* and *rhlR*, and genes involved in protein secretion, translation, and response to oxidative stress. We show that these anti-oxidant systems, AhpC-F, AhpB-TrxB2 and Dps, are critical for *P. aeruginosa* survival to reactive oxygen species and antibiotic tolerance. Considering that MvfR regulated compounds generate reactive oxygen species, this indicates a tightly regulated QS self-defense anti-poisoning system. These findings also challenge the current hierarchical regulation model of *P. aeruginosa* QS systems by revealing new interconnections between them that suggest a circular model. Moreover, they uncover a novel role for MvfR in self-defense that favors antibiotic tolerance and cell survival, further demonstrating MvfR as a highly desirable anti-virulence target.

*Pseudomonas aeruginosa* is a major nosocomial pathogen representing a critical threat for human health^1^,^2^ because of its tolerance and rapid development of resistance towards almost all current antimicrobial therapies^3^,^4^,^5^,^6^,^7^. *P. aeruginosa* acute and chronic infections are facilitated by a wide array of virulence factors, including toxins, small molecules and secondary metabolites as well as defense systems against host immunity and bacterial competitors. *P. aeruginosa* interactions with host and bacterial competitors generate environments with high levels of reactive oxygen species (ROS)^8^,^9^,^10^,^11^,^12^,^13^,^14^,^15^ that *P. aeruginosa* survives to by virtue of its multiple antioxidant systems^16^,^17^.

Most of *P. aeruginosa*’*s* virulence factors are controlled via the three major cell density dependent quorum sensing systems: LasR^18^, RhlR^19^,^20^ and MvfR (also known as PqsR)^21^,^22^,^23^,^24^. The current view is that these three systems are hierarchically connected with LasR positioned at the top of this hierarchy^25^,^26^,^27^. LasR and RhlR directly control the production of their respective activating inducers, acyl-homoserine lactones (HSL) 3-oxo-C12-HSL and C4-HSL encoded via the synthetases *lasI* and *rhlI* respectively^18^,^28^,^29^,^30^. LasR binds to 34 additional loci in *P. aeruginosa* genome, including *mvfR* and *rhlR*, and directly regulates the expression of multiple genes, including transcriptional regulators which ultimately result in the indirect modulation of more than 300 genes across the genome^31^. RhlR directly controls the expression of *rhlAB* and *rhlC* responsible for the biosynthesis of the rhamnolipid surfactants^32^,^33^ and also indirectly controls the expression of multiple genes^34^. MvfR also controls its own activity by binding and positively regulating the expression of *pqsABCDE* and *phnAB* operons that catalyze the biosynthesis of MvfR inducers and of ~60 distinct low-molecular-weight compounds^21^,^22^,^23^,^35^,^36^, including hydroxyquinolones (HAQs)^37^ and the non-HAQ molecule 2-AA^38^,^39^,^40^. Two of the most abundant HAQs (4-hydroxy-2-heptylquinoline [HHQ] and 3,4-dihydroxy-2-heptylquinoline [Pseudomonas Quinolone Signal-PQS]) bind and activate MvfR, leading to the induction of the many virulence factors that promote infection^23^,^35^,^41^,^42^,^43^. MvfR activity correlates with HHQ synthesis. Thus, an essential step of MvfR regulon activation by MvfR is the binding of MvfR protein to the *pqsABCDE* and *phnAB* operons^23^,^35^. So far, these were the only two operons to which MvfR was known to bind^22^,^35^,^44^ and the fact that MvfR is regulating the expression of 18% of *P. aeruginosa* genome^45^ was attributed to indirect effects.

The three QS systems appear to be interconnected in multiple and complex ways. RhlR and LasR QS systems both activate each other^46^. RhlR directly inhibits the expression of *pqsA* and *mvfR* by binding to their respective promoters^35^,^44^, and the MvfR regulon appears to be interconnected with RhlR via *pqsE*, the last gene of the *pqs* operon controlled by MvfR^47^. On the other hand LasR positively regulates MvfR, as it binds and induces *mvfR* expression during exponential phase^27^,^35^, with MvfR eventually becoming LasR-independent at the later stages of growth^35^. Another interconnection between the LasR and MvfR systems is that MvfR, via the *pqs* operon, controls the synthesis of the precursors of PQS and of the programmed cell death signal 2-n-heptyl-4-hydroxyquinoline-N-Oxide (HQNO)^13^, while LasR controls the enzymatic conversion of their precursors into these molecules by controlling the expression of *pqsH* and *pqsL* genes respectively^26^,^37^,^48^.

Here, our genome-wide analysis provides strong evidence that in addition to direct control of the *pqsABCDE*, *phnAB* and *mvfR genes,* MvfR may also bind to 34 additional loci across the genome of *P. aeruginosa* and fine-tune the expression of the associated genes. This work provides novel insights into the quorum sensing circuits in *P. aeruginosa* that are crucial for both pathogenesis and cell survival in deleterious environments, and its interconnection to the other *P. aeruginosa* QS systems, as well as its role in self-defense response that favors antibiotic tolerance.

## Results

### MvfR binds to and regulates the expression of multiple virulence-related loci in *P. aeruginosa* genome

**Figure 1:**
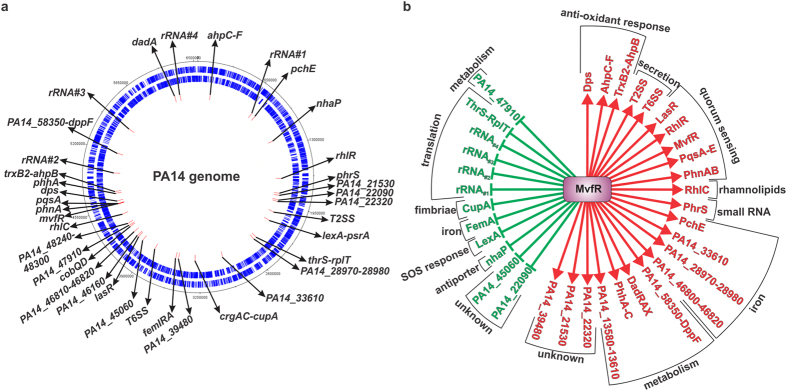
MvfR binding sites. (**a**) Localization of MvfR binding sites in PA14 genome. The first outer black circle represents the PA14 circular genome. Genes encoded on the positive strand (second circle) or negative strand (third circle) are shown in blue. Figure generated with IGV software^102^. MvfR binding sites are represented by the red rectangles (fourth circle). (**b**) Categorization of genes associated with MvfR binding sites. Positive regulation is indicated by a red arrow and negative regulation by a green flat bar.

Previous studies reported that as cell density increases MvfR regulates more genes, reaching 18% of the *P. aeruginosa* genome at the onset of stationary phase^45^. To elucidate the mode of action of MvfR on the expression of QS-controlled genes, we utilized a genome-wide approach and performed chromatin immuno-precipitation sequencing (ChIPseq) coupled with RNA sequencing (RNAseq). To fully grasp the MvfR binding dynamics, we performed this analysis at four time points corresponding to different bacterial growth stages. We used cells from early (OD_600nm_ 1.0), middle (OD_600nm_ 2.0) and late (OD_600nm_ 3.0) exponential phase as well as stationary phase (OD_600nm_ 4.0) of growth. MvfR interacting DNA was immuno-precipitated and identified by Illumina sequencing. Table 1 and Fig. 1a show that MvfR binds to 37 loci across the PA14 genome. Amongst these 37 loci, we found the expected *pqsA*, *phnA* and *mvfR* promoters, thus validating our approach. MvfR binding was also validated *in vivo* (bacterial cultures) by ChIPqPCR on some of those key loci (Supplementary Figure S1).

To correlate MvfR binding with the targeted gene expression, RNAseq studies were carried out using the parental strain PA14 and the isogenic *mvfR* mutant. Gene expression analysis of the RNAseq studies indicates that MvfR regulates 95% of the genes associated with its binding sites, with 64% being induced and 31% repressed. MvfR action on the regulation of two remaining sites is unclear (Fig. 1b, Table 1 and Supplementary Table S1).

**Figure 2:**
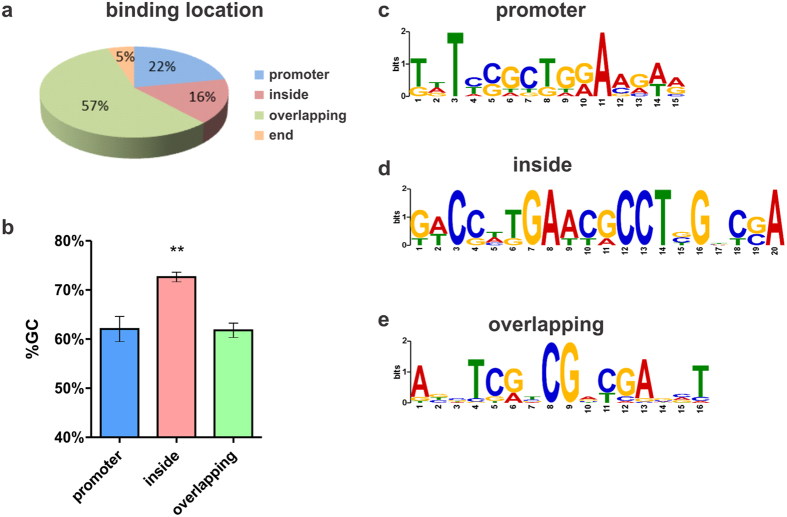
MvfR binding pattern. (**a**) Frequency of MvfR binding sites based on location. (**b**) Percentage of GC content in the DNA sequence of MvfR binding sites according to the site location, either at the promoter region (blue), inside a gene (red) or overlapping several genes (green). Statistical significance was assessed using one way ANOVA + Dunnett’s post-test. (**c–e**) MvfR binding motif identified using MEME suite^50^ according to the site location, either at the promoter region (**d**), inside a gene (**e**) or overlapping several genes (**f**).

MvfR binding to the 37 loci is either at the promoter region (22%), extends over several genes (57%), within genes (16%), or at the end of genes (5%) (Fig. 2a). Interestingly, MvfR binding sites located within genes harbor a 72% GC content, which is significantly higher than all the other MvfR binding sites (Fig. 2b). These data suggest that MvfR may exhibit different binding patterns, and potentially recognize different DNA binding motifs, consistent with the ability of LysR Type of Transcriptional Regulators (LTTR) to bind at different gene regions^49^. *In silico* analysis using MEME suite^50^ indeed shows that the predicted consensus motifs differ based on MvfR binding location (Fig. 2c–e).

Functional categorization of the genes associated in 37 loci to which MvfR binds reveals that they are mainly involved in virulence related functions, including protein secretion, quorum sensing, rhamnolipids biosynthesis and iron acquisition but also in functions related to cell metabolism, transport of small molecules, translation and response to oxidative stress (Table 1 and Fig. 1b). The following sections focus on the MvfR binding and gene expression of some of these key virulence functions.

### MvfR contributes to the induction of both RhlR and LasR QS systems

**Figure 3:**
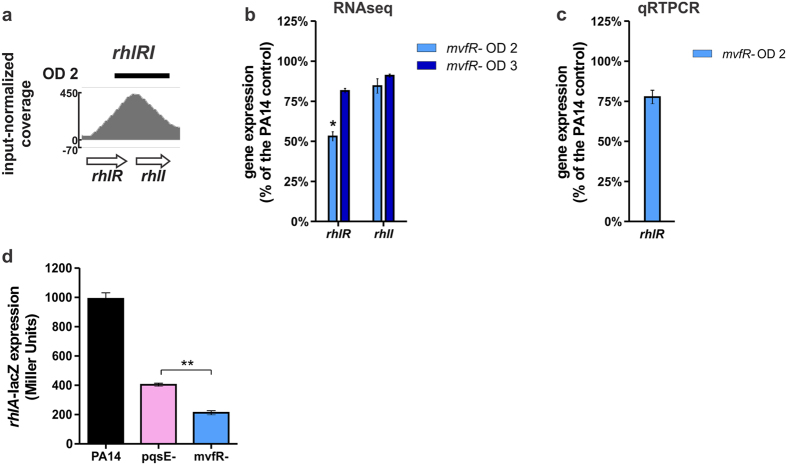
MvfR binds to and induces genes involved in the RhlR QS system. (**a**) ChIPseq analysis reveals that MvfR binds to the *rhlR-rhlI* region. The black bar above the binding intensity plot represents the peak identified using SPP peak caller. (**b**) RNAseq analysis indicates that MvfR induces the expression of *rhlR*. Light blue bar = *mvfR* mutant at OD_600nm_ 2, dark blue bar = *mvfR* mutant at OD_600nm_ 3. (**c**) qRTPCR analysis validates that MvfR induces the expression of *rhlR*. Light blue bar = *mvfR* mutant at OD_600nm_ 2. Data show the average +/− SEM of 3 independent replicates. (**d**) MvfR has an additional layer of control on *rhlA* expression in addition to PqsE as *rhlA* promoter activity is significantly lower in *mvfR* mutant than in *pqsE* mutant (p < 0.01, unpaired T test). Data show the average +/− SEM of at least 3 independent replicates.

RhlR and LasR are the two other *P. aeruginosa* main QS systems, both directly and indirectly controlling multiple virulence genes, including the MvfR QS system. Figures 3a and S1 show that MvfR binds to the *rhlR* – *rhlI* locus at OD_600nm_ 2.0. Accordingly, the expression of *rhlR* is significantly reduced in the *mvfR* mutant at OD_600nm_ 2.0 (Fig. 3b,c), suggesting another level of regulation of the RhlR QS system aside from what was previously described via PqsE^24^,^51^,^52^. Indeed, as shown in Fig. 3d, the expression of *rhlA* – directly regulated by RhlR – is significantly lower in the *mvfR* mutant than in the *pqsE* mutant (p < 0.01) supporting the existence of an additional regulatory role of MvfR on the RhlR QS system independent of PqsE.

**Figure 4:**
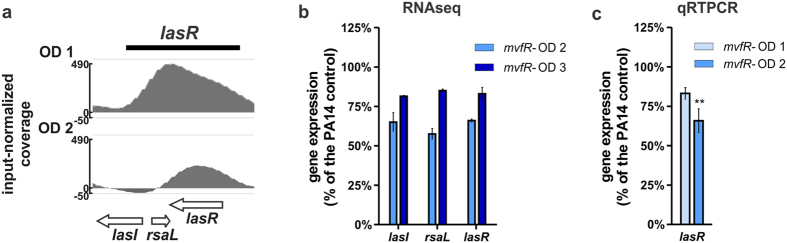
MvfR binds to and fine tunes the expression of LasR QS system genes. **(a**) ChIPseq analysis reveals that MvfR binds to *rsaL-lasR* region. The black bar above the binding intensity plots represents the peak identified using SPP peak caller. (**b**) RNAseq analysis indicates that MvfR induces the expression *lasR*, *lasI* and *rsaL*. Light blue bar = *mvfR* mutant at OD_600nm_ 2, dark blue bar = *mvfR* mutant at OD_600nm_ 3. (**c**) qRTPCR analysis validates that MvfR induces the expression of *lasR*. Faint blue bar = *mvfR* mutant at OD_600nm_ 1, light blue bar = *mvfR* mutant at OD_600nm_ 2. Data show the average +/− SEM of 3 independent replicates. Statistical significance was assessed using one way ANOVA + Dunnett’s post-test.

The ChIPseq analysis also shows MvfR binding over the region containing *lasR* and *rsaL* genes (Figs 4a and S1). Consistently, *lasI*, *rsaL* and *lasR* expression is reduced in the *mvfR* mutant, indicating that MvfR acts as an activator for this region (Fig. 4b,c). MvfR binding on these genes occurs early (OD_600nm_ 1.0) (Fig. 4a), which correlates with the early effect on the transcription of these genes (Fig. 4c).

Taken together, these data suggest a direct control of MvfR on RhlR and LasR QS systems during early and mid-exponential phase. This finding provides an interesting alternative to our current view of the hierarchical regulation of the QS systems as it rather supports a circular, interconnected regulation of the three systems.

### MvfR promotes a positive feedback loop via induction of the *phrS* small RNA

**Figure 5:**
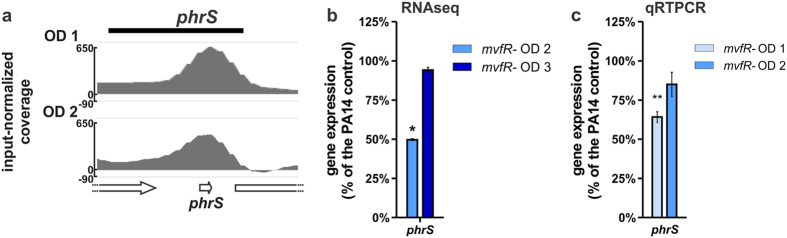
MvfR generates a positive feedback loop by binding to and inducing the small RNA PhrS. (**a**) ChIPseq analysis reveals that MvfR binds to *phrS* region. The black bar above the binding intensity plots represents the peak identified using SPP peak caller. (**b**) RNAseq analysis indicates that MvfR induces the expression of *phrS*. Light blue bar = *mvfR* mutant at OD_600nm_ 2, dark blue bar = *mvfR* mutant at OD_600nm_ 3. (**c**) qRTPCR analysis validates that MvfR induces the expression of *phrS*. Faint blue bar = *mvfR* mutant at OD_600nm_ 1, light blue bar = *mvfR* mutant at OD_600nm_ 2. Data show the average +/− SEM of 3 independent replicates. Statistical significance was assessed using one way ANOVA + Dunnett’s post-test.

*phrS* is a small RNA known to positively regulate MvfR at the post-transcriptional level leading to increased PQS and pyocyanin production, ultimately promoting *P. aeruginosa* virulence^53^. As shown in Figs 5a and S1, MvfR binds to *phrS* gene region. Accordingly, *phrS* gene expression is significantly decreased in *mvfR* mutant relative to PA14 (Fig. 5b,c). Consistent with the MvfR binding pattern to *phrS* gene (Fig. 5a and Table 1), *phrS* expression is decreased at OD_600nm_ 2.0 but not OD_600nm_ 3.0 (Fig. 5b). These data indicate that MvfR promotes a positive feedback loop via the direct induction of the *phrS* small RNA.

### MvfR induces type II and type VI protein secretion systems

**Figure 6:**
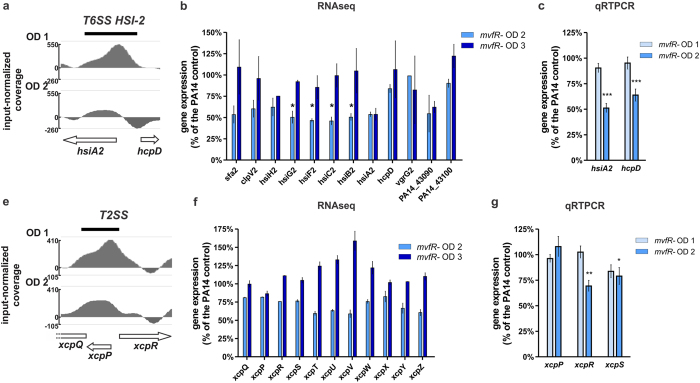
MvfR binds to and fine tunes the expression of T6SS and T2SS operons. (**a,d**) ChIPseq analysis reveals that MvfR binds to *hsiA2-hcpD* (T6SS HSI-II) and *xcpQ-xcpP-xcpR* (T2SS) regions. The black bar above the binding intensity plots represents the peak identified using SPP peak caller. (**b,e**) RNAseq analysis indicates that MvfR induces the expression of most T6SS and T2SS genes. Light blue bar = *mvfR* mutant at OD_600nm_ 2, dark blue bar = *mvfR* mutant at OD_600nm_ 3. (**c,f**) qRTPCR analysis validates that MvfR induces the expression of T6SS and T2SS genes. Faint blue bar = *mvfR* mutant at OD_600nm_ 1, light blue bar = *mvfR* mutant at OD_600nm_ 2. Data show the average +/− SEM of 3 independent replicates. Statistical significance was assessed using one way ANOVA + Dunnett’s post-test.

*P. aeruginosa* secretes important toxins (i.e. elastase, exotoxins, phospholipases) via the type II secretion system (T2SS) into the extracellular environment^54^. The type VI secretion system (T6SS) is mostly involved in antagonistic interactions with bacterial competitors^55^. We have shown previously that MvfR impacts the transcription of T6SS^42^ and T2SS and the level of secreted exoproducts of the T2SS^22^,^45^. MvfR positively regulated the transcription of T6SS HSI-II genes, while surprisingly they were reported to be unchanged in a *pqsE* mutant^42^, suggesting that MvfR is the major contributor for this regulation. This notion is supported by the fact that MvfR binds over *hsiA2* and *hcpD* of T6SS HSI-II (Figs 6a and S1). Gene expression studies in the *mvfR* mutant reveal that MvfR induces the expression of most T6S HSI-II genes at OD_600nm_ 2.0 but not at OD_600nm_ 3.0 (Fig. 6b,c). As such, they are consistent with the impact of MvfR binding during early exponential phase and the absence of MvfR binding later (Fig. 6a and Table 1), and imply a direct role for MvfR in the expression of this locus at the early and mid-exponential stage of growth.

Figures 6d and S1 show that MvfR also binds to T2SS genes *xcpQ*, *xcpP* and *xcpR*, key genes that confer functionality to this system for the secretion of several virulence exoproducts^54^,^56^. Accordingly, the expression of most T2SS genes is reduced in *mvfR* mutant at OD_600nm_ 2.0 but no reduced expression of these genes is observed at OD_600nm _= 3, consistent with the absence of detectable MvfR binding at these later ODs (Fig. 6e,f and Table 1). Together, these data suggest that MvfR directly contributes to the regulation of key genes of T6SS HSI-II and T2SS at the early stages of *P. aeruginosa* growth.

### MvfR binds to five translation-related loci and negatively impacts *P. aeruginosa* translation

**Figure 7:**
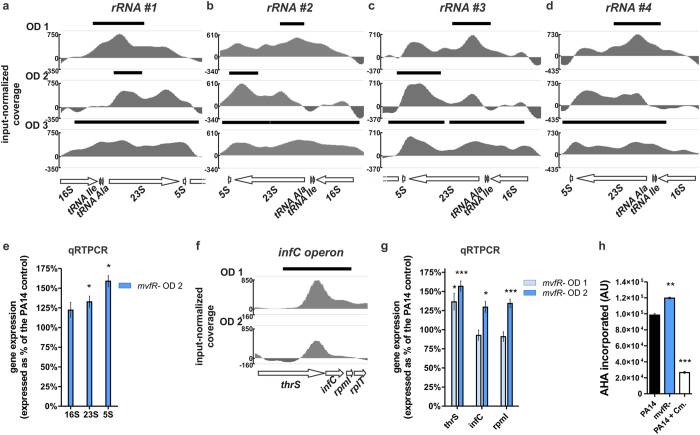
MvfR slows down translation activity by binding to and negatively regulating translation related genes. (**a–d,f**) ChIPseq analysis reveals that MvfR binds to the 4 rRNA regions – *rRNA* #1 (**a**), *rRNA* #2 (**b**), *rRNA* #3 (**c**) and *rRNA* #4 (**d**) – as well as the *infC* operon (**f**). The black bar above the binding intensity plots represents the peak identified using SPP peak caller. (**e,g**) qRTPCR analysis indicates that MvfR represses the expression of 16S, 23S and 5S rRNAs as well as *thrS*, *infC*, and *rpmI*. Faint blue bar = *mvfR* mutant at OD_600nm_ 1, light blue bar = *mvfR* mutant at OD_600nm_ 2. Data show the average +/− SEM of 3 independent replicates. Statistical significance was assessed using unpaired T test for *rRNA* genes and one way ANOVA + Dunnett’s post-test for *infC* operon genes. (**h**) MvfR inhibits the translation activity of *P. aeruginosa* as reflected by the measurement of newly synthetized proteins using the labeled amino-acid alanine AHA (L-azidohomoalanine). The translation inhibitor chloramphenicol (15 mg/L) was used as a control. Data show the average +/− SEM of three independent replicates. Statistical significance was assessed using one way ANOVA + Dunnett’s post-test.

We reported previously that the MvfR negatively modulates the expression of *P. aeruginosa* protein translation genes^6^. However, the underlying molecular mechanism of this negative regulation is unclear. Figure 7 shows that MvfR binds to and controls the expression of five translation related loci. Binding of MvfR extends along an entire region comprised of genes encoding 5S, 16S, 23S rRNAs and two tRNAs. MvfR binding is present during OD_600nm_ 1.0–3.0 in all four repeats of this region throughout the PA14 genome (Fig. 7a–d). In addition, MvfR binds to the translation initiation factor IF-3 encoding gene, *infC*, which is the second gene of an operon also encoding the translation-related genes, *thrS*, *rpmL* and *rplT* (Fig. 7f). Expanding on what we previously reported^6^, we show here that the expression of 16S, 23S and 5S rRNAs is increased in the *mvfR* mutant (Fig. 7e). Moreover, the expression of *thrS*, *infC* and *rpmI* genes is also significantly increased (Fig. 7g), indicating that MvfR acts as a negative regulator of this set of translation-related genes. Consistently, *P. aeruginosa* translational activity, measured by the incorporation of L-azidohomoalanine into proteins^57^, is significantly increased in the *mvfR* mutant compared to PA14 (Fig. 7h), validating the role of MvfR in translation inhibition. Overall, these findings imply a direct negative fine-tuning action of QS on *P. aeruginosa* translation machinery via MvfR binding to *rRNA* genes and *infC* operon.

### MvfR induction of antioxidant genes contributes to antibiotic tolerance

**Figure 8:**
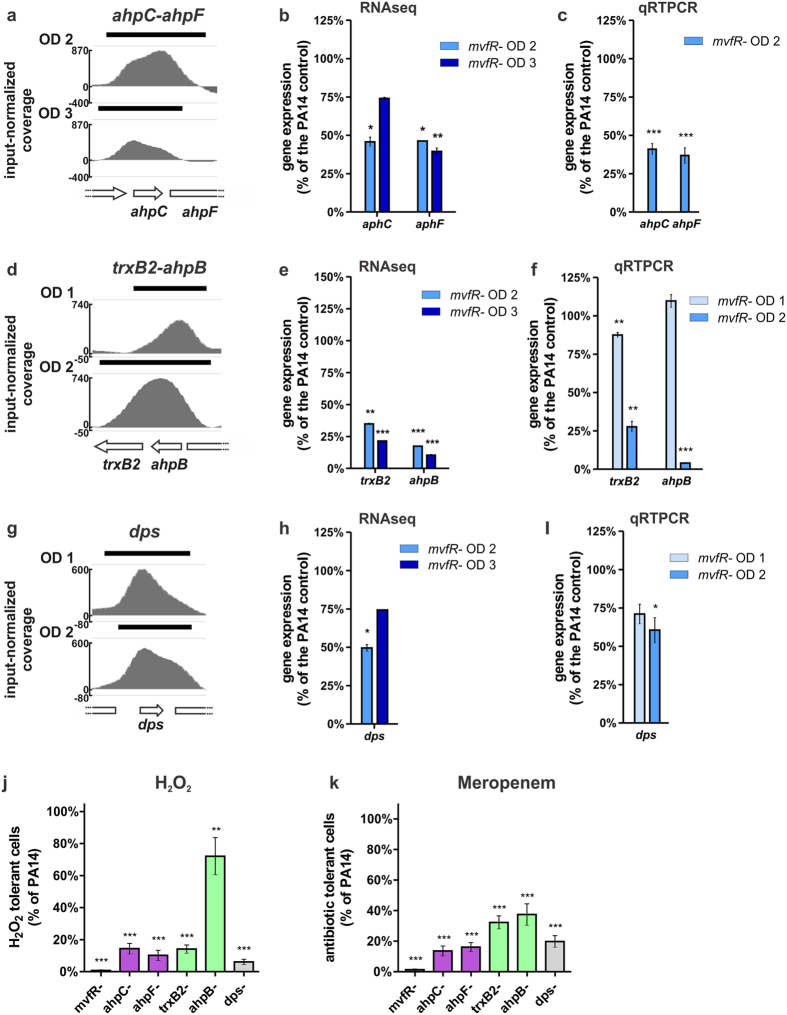
MvfR binds to and positively regulates genes involved in response to oxidative stress which leads to tolerance of H_2_O_2_ and antibiotic. (**a,d,g**) ChIPseq analysis reveals that MvfR binds to *ahpC-ahpF, trxB2-ahpB and dps regions*. The black bar above the binding intensity plots represents the peak identified using SPP peak caller. (**b,e,h**) RNAseq analysis indicates that MvfR induces the expression of *ahpC*, *ahpF, trxB2, ahpB and dps*. Light blue bar = *mvfR* mutant at OD_600nm_ 2, dark blue bar = *mvfR* mutant at OD_600nm_ 3. (**c,f,g**) qRTPCR analysis validates that MvfR induces the expression of *ahpC*, *ahpF*, *trxB2*, *ahpB* and *dps*. Faint blue bar = *mvfR* mutant at OD_600nm_ 1, light blue bar = *mvfR* mutant at OD_600nm_ 2. Data show the average +/− SEM of 3 independent replicates. Statistical significance was assessed using one way ANOVA + Dunnett’s post-test. (**i,j**) *mvfR* mutant (red) as well as *ahpC* and *ahpF* mutants (purple), *trxB2* and *ahpB* mutants (green), and *dps* mutant (grey) are more sensitive than PA14 to hydrogen peroxide (**i**) or the β-lactam antibiotic Meropenem (**j**). The survival fraction of PA14 control after H_2_O_2_ or Meropenem treatment is of 6.8 × 10^−4^ or 7.1 × 10^−6^ respectively. Data show the average +/− SEM of at least 3 independent replicates. Statistical significance was assessed using one way ANOVA + Dunnett’s post-test.

Perhaps one the most interesting sets of MvfR binding loci are those related to the oxidative stress response. As shown in Figs 8a,d,g and S1, MvfR binds to *ahpC*-*ahpF*, *trxB2*-*ahpB* and *dps* regions. Accordingly, the expression of all those genes is significantly reduced in the *mvfR* mutant (Fig. 8b,c,e–h). In corroboration, our previous microarray expression studies also showed that the expression of these genes is downregulated in the presence of MvfR QS system inhibitors^42^. The alkyl-hydroxyperoxidases AhpC, AhpF and AhpB, as well as the threonine reductase TrxB2, and the Dps protein are involved in the detoxification of hydrogen peroxide and organic peroxides^17^,^58^,^59^,^60^ and iron oxidation and sequestration. These proteins ultimately prevent the generation of lethal Fenton reaction derived hydroxyl radicals^61^,^62^. Consistently, we observed that AhpC, AhpF, TrxB2, AhpB and Dps allow tolerance to hydrogen peroxide given that mutants in those genes are 1.4 to 17 times more sensitive to H_2_O_2_ than PA14 (Fig. 8i). The *mvfR* mutant is also significantly more sensitive to H_2_O_2_ than PA14 (Fig. 8i), thereby supporting the relevance of MvfR in response to oxidative stress.

Importantly, antioxidant defenses are known to play a protective role against antibiotics via their ROS suppressing activities^63^,^64^,^65^,^66^,^67^,^68^,^69^. We previously described the importance of MvfR in antibiotic tolerance^6^,^43^,^70^, and here we asked whether the detoxification abilities of AhpC, AhpF, TrxB2, AhpB and Dps may contribute to this phenomenon. As shown in Fig. 8j, *ahpC, ahpF, trxB2, ahpB* and *dps* mutants are 2.7 to 7.7 times more sensitive to the β-lactam antibiotic Meropenem than the parental PA14 strain, indicating that direct MvfR control of AhpC-F, TrxB2-AhpB and Dps contributes to antibiotic tolerance.

## Discussion

This study provides novel insights in the understanding of MvfR role in the complex regulation of QS and pathogenesis in *P. aeruginosa*. Indeed, our data strongly suggest that MvfR acts as a direct modulator of key *P. aeruginosa* virulence systems beyond the biosynthetic operons *pqsABCDE* and *phnAB* in line with its original nomenclature as Multiple Virulence Factor Regulator (MvfR)^22^. Moreover, this work permits us to better comprehend the regulation of QS and challenges the current hierarchical view that LasR is upstream of MvfR and that MvfR controls QS virulence only indirectly through the transcriptional control of the *pqsABCDE* operon^24^,^51^,^52^.

Previous studies have shown the broad impact of MvfR in the transcriptional regulation of up to 18% of *P. aeruginosa* genes^45^. However, the possibility of MvfR direct regulation for some of these genes has been overshadowed by the role of the HAQs and PqsE in virulence^26^,^47^,^51^,^52^,^71^,^72^. It is noteworthy that PQS is dispensable for virulence since *pqsH* mutation does not attenuate virulence in mice^23^. The suggested direct transcriptional regulation of MvfR on *P. aeruginosa* genes described here could account for the already established indirect regulation working via PqsE and HHQ/PQS^26^,^47^,^51^,^52^,^71^,^72^. Even though the 34 loci identified represent only a fraction of MvfR regulated genes, they are nonetheless relevant because they contain specific virulence factors as well as global regulators of virulence, including the two other major QS regulators LasR and RhlR. The increased number of genes regulated by MvfR at late exponential and stationary phases of growth most likely stems from direct MvfR regulation of key cellular, metabolic and QS regulatory functions at earlier stages of growth.

We noticed that MvfR exhibits different binding patterns and recognizes different DNA binding motifs. The predicted MvfR consensus binding motifs in both promoter regions and regions overlapping several genes share the classical LTTR TN_x_A palindrome structure^49^. Tomtom analysis^73^ indicates that the predicted consensus motif for sites located inside genes resembles the DNA binding motif of RutR, an *E. coli* transcriptional regulator known to bind specifically within genes^74^ which is consistent with this group of MvfR binding sites. Such intragenic binding and the ability of MvfR to act as negative and positive regulator have previously been described with other members of the LTTR family^49^,^75^. It is important to note that the crosslinking step inherent to all ChIP procedures may lead to the capture of protein-protein interactions and potentially to false-positive binding sites. Future in depth mechanistic studies focusing on MvfR binding patterns will allow us to conclusively demonstrate the MvfR direct regulation of the loci identified and obtain a better understanding of how and why MvfR interacts with DNA in this fashion.

**Figure 9:**
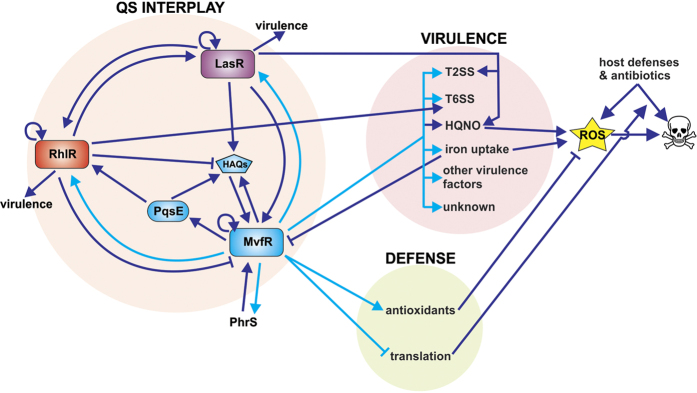
Proposed model of the role of MvfR direct regulation on QS interplay, virulence and defense. This proposed model figure focuses on MvfR direct regulation and the systems that feedback to it either positively (arrow) or negatively (bar). Dark blue arrows/bars represent connections previously described in the literature whereas light blue arrows/bars represent new connections based on this study.

The longitudinal interrogation of MvfR binding during *P. aeruginosa* growth supports an interconnected regulation pathway for the three major QS systems, as MvfR is likely involved in the direct modulation of both LasR and RhlR QS systems, which were both previously shown to regulate the MvfR QS system^27^,^35^,^44^. The data presented here challenge the hierarchical regulation model of *P. aeruginosa* QS systems and introduce a circular regulation model (see proposed model in Fig. 9). Intriguingly, MvfR binding to and modulation of *lasR* and *rhlR* occurs at early and mid-exponential phase (OD_600nm_ 1.0 and 2.0) raising the question of why this timing is important. At late exponential and stationary phase *rhlR* expression and C4-HSL levels max out^45^,^46^ and RhlR binds to *mvfR* and *pqsA* promoters acting as a direct repressor of the MvfR QS system^35^,^44^, promoting a tight negative auto-regulatory loop. In contrast, MvfR binding to *lasR* likely feeds back into the MvfR QS circuitry by inducing *mvfR* expression^27^,^35^, and the PqsH-mediated conversion of HHQ into PQS increases MvfR activity^43^,^48^. Two other suggested positive feedback loops occurring at the earlier stages of growth result from MvfR binding to itself and *phrS* small RNA, which was previously described as a post-transcriptional activator of MvfR^53^. Until now, *phrS* induction was reported to take place at the stationary growth phase as a result of its control by the transcriptional regulator ANR under hypoxic conditions^53^. Here, our data indicate that MvfR could directly induce *phrS* expression during early and mid-exponential phase, before ANR and hypoxia come into play. These two suggested positive feedback loops may benefit the MvfR QS system by rapidly increasing MvfR levels and generating functional intracellular levels of MvfR ligands before quorum levels are reached in the global cell population.

Another interesting regulation identified in this study is related to iron uptake and homeostasis. Indeed, we notice MvfR binding to and inducing the two siderophores, pyochelin and pyoverdin, and the putative iron transporters PA14_28970-28980 and PA14_46810-46820 (Table 1 and Supplementary Table S1). Iron is an essential cofactor for a wide variety of cellular processes but is especially scarce during host infection due to nutritional immunity^76^,^77^,^78^. However, excessive iron levels can lead to cellular damage and ultimately cell death as iron catalyzes ROS production via the Fenton reaction^62^,^79^. Therefore, iron homeostasis is critical. Once intracellular iron levels are high, uptake systems and their regulators, including MvfR, are repressed by the Fur transcriptional regulator^80^. This negative feedback loop also turns down the production of all other ROS-inducing systems under MvfR control (i.e. Pyocyanin, HQNO)^8^,^13^.

Even though the iron uptake and ROS producing systems can be deleterious for *P. aeruginosa*, they are nonetheless critical for survival, colonization and competition with other bacterial species^8^,^9^,^10^,^81^,^82^. Therefore, the ability of *P. aeruginosa* to survive when these systems are active offers an important selective advantage. Here we show that MvfR enhances protection against ROS that can be produced by these systems via binding to and inducing the expression of the anti-oxidant defense systems AhpC-F, AhpB-TrxB2 and Dps. These antioxidant proteins are known to limit ROS production by reducing the levels of hydrogen peroxide and iron available as substrates for the Fenton reaction^59^,^61^,^62^,^83^. As such MvfR modulation of these antioxidant genes may allow cells to survive the production of oxidative toxins (see proposed model in Fig. 9). This self-protective “toxin/anti-toxin” system is reminiscent of antibiotic producing bacteria that require a self-resistance mechanism to avoid committing suicide^84^,^85^. Protection against self-poisoning is not the only role of these antioxidants, as we show here that they contribute to antibiotic tolerance. MvfR’s ability to induce their expression provides an explanation for the previously unknown molecular mechanism of MvfR-mediated antibiotic tolerance. In addition to inducing antioxidant systems, we also show that MvfR binds to translation-related loci and represses translation. Since antibiotic tolerant cells are known to exhibit reduced protein synthesis and metabolic rates^4^,^5^,^86^,^87^, MvfR action on translation may also contribute to antibiotic tolerance (see proposed model in Fig. 9).

Overall, our data suggest that MvfR directly controls the expression of multiple virulence factors and plays a central role in the *P. aeruginosa* QS interplay as well as in antibiotic tolerance via the regulation of multiple antioxidant systems. This highlights the importance of MvfR as a critical virulence determinant and reinforces its potential as a highly desirable drug target candidate 42,43,88,89,90,91.

## Methods

### Bacterial strains, plasmids, growth conditions

UCBPP-PA14 (PA14) is a *P. aeruginosa* human clinical isolate^92^. All mutant strains including *mvfR-*22, *pqsA-*21 and *pqsE-*47 are isogenic to UCBPP-PA14. pECP60 plasmid containing the *rhlA-lacZ* reporter system was described previously^45^,^93^. Unless noted otherwise, all bacterial strains were grown in 5 mL LB Lenox medium (Fisher Scientific) at 37 °C under 200 rpm orbital shaking using glass tubes (VWR). To generate pJN-mvfR-FLAG plasmid expressing C-terminally-FLAG tagged MvfR, the 657-bp upstream region and *mvfR* coding region with C-terminal FLAG was amplified by PCR and then cloned into pJN105 treated with EcoR1 and Xho1 as described in^94^. The resulting plasmid was transformed into *mvfR-* cells. The mvfR- strain carrying pJN-mvfR-FLAG was grown in the presence of 15 μg/mL Gentamycin.

### ChIPseq

5 ml cultures were inoculated at OD_600nm_ 0.1, and grown at 37 °C 200 rpm in LB Lenox + 15 μg/mL Gentamycin. At OD_600nm_ 1, 2, 3 and 4, cells were washed in fresh LB Lenox then pelleted and stored at −80 °C. The ChIP experiment was performed as described in^43^,^95^ using anti-FLAG M2 magnetic beads (Sigma). Library construction and Illumina DNA sequencing was performed by htSEQ (Seattle, WA). The sequence aligner BWA^96^ was used to map sequencing reads to a UCBPP-PA14 fasta reference genome file (RefSeq May 24, 2010). Peaks for samples at OD_600nm_ 1, 2, 3 and 4 were identified using the ChipSeq peak caller SPP^97^ with OD3_input as the reference input. Peaks were filtered at a Z_score = 9 in order to limit the probability of false positives. It is noteworthy that pH changes over time during growth have the potential to affect iron bioavailability, and may also drive changes in the physiology of the microbe that could affect binding.

### ChIP qPCR

We validated the ChIPseq findings via ChIP qPCR and not via EMSA studies because this approach allows us to interrogate MvfR binding in live cells where all biologically relevant components are present, and also to bypass the known difficulties to consistently purify full-length MvfR protein^88^,^98^. Samples were prepared as described in the ChIPseq section above. qPCR was then performed using primers designed to be in the middle of MvfR binding sites using the Primer 3 tool (http://bioinfo.ut.ee/primer3-0.4.0/). The primer sequences for each site are listed in Supplementary Table S2. Quantitative PCR was performed using Brilliant II SYBR Green QPCR Master Mix (Stratagene) as in^43^,^47^ with a Mx3005P qPCR machine (Stratagene). Data were analyzed using the percent input method and using *rpoD* as a negative control as in ref. 43.

### RNAseq

PA14 and *mvfR-* cells were grown in LB Lenox at 37 °C 200 rpm until OD_600nm_ 2 or OD_600nm_ 3. Duplicates of each culture were processed for RNA extraction using the RNeasy kit (QIAGEN) and DNAse treatment was performed with the TURBO DNA-free kit (Thermo-Fisher). RNA samples were then subjected to rRNA depletion using RiboZero (Epicentre) followed by the construction of next-generation sequencing libraries using NEBNext Ultra Directional RNA Library Prep Kit (New England Biolabs). These libraries were sequenced on Illumina HiSeq 2500 instrument resulting in approximately 8.8 million reads per sample on average. Subsequent to alignment using BWA^96^, read counts for individual transcripts were produced with HTSEQ^99^, based on UCBPP-PA14 transcriptome annotation (NC_002516). The estimation of expression values and detection of differentially expressed transcripts was performed using EdgeR^100^. Statistical significance was assessed using false discovery rate.

### qRT-PCR

RNA samples were prepared as described above for RNAseq. cDNA was generated by RT-PCR using Superscript III First-Strand kit (Invitrogen) according to manufacturer’s instructions. Specific primers were designed using Primer3. Primers used are described in Supplementary Table S2. RpoD expression was used as a reference gene as described in^47^,^101^. Quantitative PCR was performed using Brilliant II SYBR Green QPCR Master Mix (Stratagene) as in^43^,^47^ with a Mx3005P qPCR machine (Stratagene).

### Translation activity assay

Translation activity was determined using Click chemistry as described in^57^ with modifications. PA14 and *mvfR-* cells were grown in LB Lenox at 37 °C 200 rpm overnight from a frozen stock then washed and resuspended in M9 media to OD_600nm_ 0.1. Cells were grown for 5 h at 37 °C 200 rpm then 15 μg/mL chloramphenicol (or 10% ethanol vehicle) together with 1 μM Click-iT AHA L-azidohomoalanine (Life Technologies) were added for 45 minutes to the growing cultures. Cells were then pelleted and fixed in 4% PFA for 1 hour then washed in PBS. Cells were then lysed by sonication in 1% SDS 50 mM Tris-HCl pH8. Labelled solubilized proteins were subjected to reaction with Click-iT Protein Reaction Buffer kit (Life Technologies) containing 4 mM tetramethylrhodamine (TAMRA) alkyne (Life Technologies) according to manufacturer’s instructions. Proteins were then extracted by the methanol-chloroform method and washed twice in methanol as described in ref. 57. 2 μL of each extracted protein sample was finally dotted on a nitrocellulose membrane and imaged using 534 nm excitation (green)/607 nm emission (orange) filter in a FluorChem M imaging device (Protein Simple). Signal was then processed using ImageJ software in order to quantify AHA labeled proteins, representing nascent protein synthesis.

### β-galactosidase gene reporter assay

PA14 and *mvfR-* cells containing pECP60 plasmid were grown in presence of 300 μg/mL Carbenicillin to maintain the plasmid. At OD_600nm_ 2, 20 μL of cells were collected and incubated with 80 μL of permeabilization solution (100 mM Na_2_HPO_4_, 20 mM KCl, 2 mM MgSO_4_, 0.8 mg/mL CTAB, 0.4 mg/mL sodium deoxycholate and 5.4 μL/mL β-mercaptoethanol) for 30 min at 37 °C. Then 600 μL of substrate solution was added (60 mM Na_2_HPO_4_, 40 mM NaH_2_PO_4_, 1 mg/mL ONPG and 2.7 μL/mL β-mercaptoethanol) and incubated at 37 °C until yellow color was detected. 700 μL of Stop solution (1M Na_2_CO_3_) was finally added to block the reaction and OD_420nm_ was measured. Miller units were calculated as follows: 1000 × OD_420nm_/(OD_600nm _× 0.02 mL × reaction time). More details on this protocol can be found at (http://openwetware.org/wiki/Beta-Galactosidase_Assay_%28A_better_Miller%29).

### Tolerance to H_2_O_2_ or Meropenem

PA14, *mvfR-*, *aphC-*, *ahpF-*, *trxB2-*, *ahpB-* and *dps-* cells were grown at 37 °C 200 rpm in LB Lenox media until mid-exponential phase (OD_600nm_ 2) then exposed to 300 mM hydrogen peroxide (H_2_O_2_) for 1 hour under the same incubating conditions. Before (t = 0) and after (t = 1 h) H_2_O_2_ addition, a 100 μL sample of each culture was collected, diluted and plated on LB agar plates to quantify the total number of bacteria (t = 0) and the surviving bacteria (t = 1 h). Colony forming units (CFUs) were counted after 24 h incubation at 37 °C. Tolerance to Meropenem was assessed the same way except the cells were grown in 1% TSB media and the killing was performed for 24 hours in presence of 10 μg/mL Meropenem (Sandoz, USA).

### Statistical analyses

Statistical significance was assessed using unpaired T test or One Way ANOVA + Dunnett’s post-test in the case of multiple comparisons, as appropriate and indicated in the figure legends.

## Additional Information

**How to cite this article**: Maura, D. *et al.* Evidence for Direct Control of Virulence and Defense Gene Circuits by the *Pseudomonas aeruginosa* Quorum Sensing Regulator, MvfR. *Sci. Rep.* **6**, 34083; doi: 10.1038/srep34083 (2016).

## Supplementary Material

Supplementary Information

## Figures and Tables

**Table 1:** List of MvfR binding sites, their associated gene function and regulation.

		Regulated gene(s)	Function	MvfR binding (Input normalized coverage)				Locus	Regulation byMvfR
Coordinates										OD 1	OD 2	OD 3	OD 4
155,548	157,602	*ahpC-ahpF*	response to oxidative stress		868	467		over.	+
733,577	738,665	*16S, 23S, 5S & tRNA*	translation (rRNA #1)	748	504	503		over.	−
788,364	789,806	*pyochelin operon*	siderophore pyochelin		375			ins.	+
1,170,619	1,171,875	*PA14_13580-13610 operonnhaP*	ABC transporterNa+/H+ antiporter		723			prom.	+
									−
1,651,474	1,652,473	*rhlR*	rhlR QS system		436			end	+
1,840,873	1,842,123	*phrS*	small RNA	643				over.	+
1,866,043	1,868,058	*PA14_21530*	unknown	447	548			over.	+
1,923,884	1,925,012	*PA14_22090*	unknown	586				prom.	−
1,943,865	1,944,935	*PA14_22320*	unknown	381				over.	+
2,083,830	2,084,914	*T2SS operons*	type 2 secretion system	408				over.	+
2,201,076	2,202,455	*lexApsrA*	SOS responsesigma factor	497				prom.	−
									+
2,467,980	2,469,951	*infC operon*	translation (initiation factor, tRNA and ribosomal proteins)	821			277	over.	−
2,504,998	2,506,157	*PA14_28970 & 28980*	putative iron transporter				107	over.	+
2,956,881	2,958,001	*pyoverdine operon*	siderophore pyoverdine				106	ins.	+
3,303,526	3,304,565	*crgAC & cupA operon*	cupA fimbriae				100	end	−
3,513,100	3,515,095	*PA14_39480*	unknown	844			232	ins.	+
3,547,291	3,548,339	*femIRA*	iron transpoter				77	prom.	+
3,830,327	3,831,831	*T6SS locus 2 operons*	type 6 secretion system	545				prom.	+
4,019,224	4,020,417	*PA14_45060*	putative urea transporter				94	over.	−
4,084,785	4,086,308	*lasR*	lasR QS system	490				over.	+
4,104,672	4,106,305	*PA14_46160*	unknown	536	493			over.	+
4,167,308	4,168,950	*PA14_46810-46820*	putative iron transporter	293			77	over.	+
4,246,274	4,247,621	*cobalamine operon*	cobalamine biosynthesis				86	ins.	?
4,263,263	4,264,333	*PA14_47910*	ABC transporter (arabinose)				99	ins.	−
4,294,736	4,295,982	*PA14_48240-48300*	putative antibiotic efflux pump				103	ins.	?
4,425,759	4,427,304	*PA14_49750 & rhlC*	rhamnolipids biosynthesis				119	ins.	+
4,561,261	4,564,718	*mvfR*	MvfR QS system	6,439	5,101		1,313	over.	+
4,565,212	4,566,614	*phnAB operon*	MvfR QS system		378			prom.	+
4,570,486	4,572,591	*pqsABCDE operon*	MvfR QS system	1,083	2,118	647	254	prom.	+
4,602,850	4,604,494	*dps*	response to oxidative stress	596	526			over.	+
4,698,069	4,699,401	*phhABC operon*	phenylalanine catabolism	338	376			over.	+
4,722,947	4,725,071	*trxB2 & ahpB*	response to oxidative stress	497	736			over.	+
4,953,754	4,957,408	*5S, 23S, tRNAs, 16S*	translation (rRNA #2)	516	606	422		over.	−
5,200,388	5,201,551	*psdR*	repressor of ABC transporter dppA-F (arginine)		525			prom.	−
5,535,420	5,540,989	*5S 23S tRNAs 16S*	translation (rRNA #3)	672	702	596		over.	−
6,245,833	6,247,899	*dadA*	D-alanine metabolism	1,734	2,081	1,043		over.	+
6,312,205	6,316,455	*5S 23S tRNAs 16S*	translation (rRNA #4)	725		420		over.	−

over. = binding site is overlapping several genes, ins. = binding site is inside a gene, prom. = binding site is in a promoter region, end = binding site is at the end of a gene, + = positive regulation, − = negative regulation, ? = unclear regulation. For more details on gene regulation, see Supplementary Table S1.
