# Supplementary material for: Evidence for Direct Control of Virulence and Defense Gene Circuits by the Pseudomonas aeruginosa Quorum Sensing Regulator, MvfR
Source: Sci Rep. 2016 Sep 28;6:34083. doi: 10.1038/srep34083 (PMC5039717; doi:10.1038/srep34083)
Supplement: Supplementary Information [file srep34083-s1.pdf]

Evidence for Direct Control of Virulence and Defense Gene Circuits by the *Pseudomonas aeruginosa* Quorum Sensing Regulator, MvfR

Damien Maura[a,b,c], Ronen Hazan[a,b,c,d], Tomoe Kitao[a,b,c], Alicia E. Ballok[a,b,c] and Laurence G. Rahme[a,b,c]*

[a]Department of Surgery, [b]Department of Microbiology and Immunobiology, Harvard Medical School and Massachusetts General Hospital, [c]Shriners Hospitals for Children Boston, Boston, 02114, Massachusetts, USA;, [d]Institute of Dental Sciences and School of Dental Medicine, Hebrew University, Jerusalem P.O.B 12272, Israel 91120

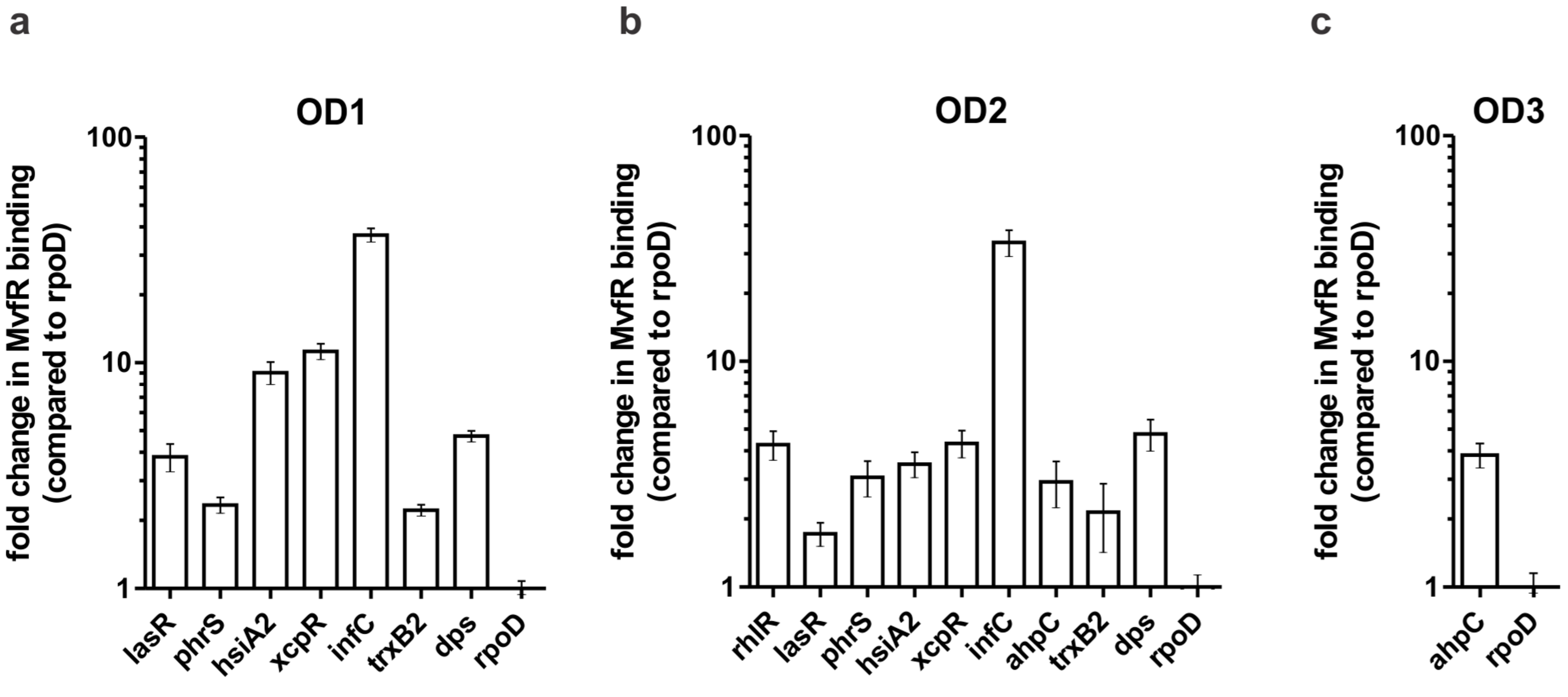

a
b
c
OD1
OD2
OD3
fold change in MvfR binding (compared to rpoD)
100
10
1
lasR
phrS
hsiA2
xcpR
infC
trxB2
dps
rpoD
rhlR
ahpC

Supplementary Figure S1: Validation by ChIPqPCR of the MvfR binding sites found in the ChIPseq

ChIPqPCR analysis validates that MvfR is binding to *rhlR*, *lasR*, *phrS*, *hsiA2*, *xcpR*, *infC*, *ahpC*, *trxB2* and *dps* compared to the negative control *rpoD*. Data show the average +/- SEM of 3 independent replicates.

MvfR binding site #	function	PA14#	gene name	regulation	% expression versus WT mvfR- OD2	% expression versus WT mvfR- OD3	p value mvfR- OD2	p value mvfR- OD3
1	antioxidant	PA14_01710	*ahpC*	positive	45.6%	74.0%	0.0163	0.3613
		PA14_01720	*ahpF*		46.3%	39.5%	0.0184	0.0046
2	rRNA #1	PA14_08570	*16S rRNA*	negative (see RTqPCR)	na.	na.	na.	na.
		PA14_08580	*tRNA-Ile*		na.	na.	na.	na.
		PA14_08590	*tRNA-Ala*		na.	na.	na.	na.
		PA14_08600	*23S rRNA*		na.	na.	na.	na.
		PA14_08610	*5S rRNA*		na.	na.	na.	na.
3	pyochelin	PA14_09270	*pchE*	positive	1.3%	2.0%	8.17E-27	2.10E-23
		PA14_09280	*pchF*		1.1%	2.1%	4.08E-28	3.28E-23
		PA14_09290	*pchG*		1.2%	2.0%	3.45E-26	1.10E-22
		PA14_09300	*pchI*		1.3%	1.2%	4.57E-26	1.56E-26
		PA14_09320	*pchJ*		0.9%	0.9%	1.02E-28	1.66E-28
4	ABC transporter	PA14_13580	nd.	positive	55.7%	103.4%	0.0744	0.8998
		PA14_13590	nd.		64.4%	124.1%	0.1921	0.4928
		PA14_13600	nd.		74.1%	131.6%	0.3724	0.3831
		PA14_13610	nd.		135.3%	192.0%	0.3674	0.0471
	cation transporter	PA14_13620	*nhaP*	negative	212.8%	93.7%	0.0264	0.8753
5	RhlR QS system	PA14_19100	*rhlA*	positive	42.9%	79.5%	0.0098	0.4894
		PA14_19110	*rhlB*		36.1%	77.7%	0.0019	0.4445
		PA14_19120	*rhlR*		52.8%	81.5%	0.0504	0.5384
		PA14_19130	*rhlI*		84.5%	90.6%	0.6154	0.7775
6	small RNA	PA14_21260	*phrS*	positive	49.7%	94.1%	0.0360	0.8752
7	unknown	PA14_21530	nd.	positive	24.3%	17.3%	4.43E-05	3.22E-07
8	unknown	PA14_22090	nd.	negative	211.6%	75.3%	0.0709	0.4699
9	unknown	PA14_22320	nd.	positive	12.6%	2.3%	5.13E-04	6.15E-19
10	T2SS	PA14_23970	*xcpQ*	positive	80.9%	99.6%	0.5210	0.9923
		PA14_23980	*xcpP*		81.6%	86.3%	0.5491	0.6684
		PA14_23990	*xcpR*		75.6%	110.9%	0.3943	0.7293
		PA14_24010	*xcpS*		76.3%	104.6%	0.4136	0.8727
		PA14_24020	*xcpT*		59.3%	124.3%	0.1119	0.4886
		PA14_24040	*xcpU*		63.2%	132.7%	0.1649	0.3724
		PA14_24050	*xcpV*		58.7%	158.7%	0.1151	0.1622
		PA14_24060	*xcpW*		75.6%	121.8%	0.4067	0.5299
		PA14_24070	*xcpX*		82.4%	101.7%	0.5730	0.9412
		PA14_24080	*xcpY*		66.3%	102.9%	0.2119	0.9138
		PA14_24100	*xcpZ*		60.5%	110.0%	0.1304	0.7542
11	SOS response	PA14_25160	*lexA*	negative	176.0%	139.4%	0.0858	0.3027
	sigma factor	PA14_25180	*prsA*	positive	56.8%	84.0%	0.0828	0.6044
12	translation	PA14_28650	*thrS*	negative (see RTqPCR)	na.	na.	na.	na.
		PA14_28660	*infC*		na.	na.	na.	na.
		PA14_28670	*rpmL*		na.	na.	na.	na.
		PA14_28680	*rplT*		na.	na.	na.	na.
13	putative iron transporter	PA14_28970	nd.	positive	66.2%	15.8%	0.5864	1.82E-05
		PA14_28980	nd.		188.0%	22.3%	0.2344	1.03E-04
14	pyoverdine	PA14_33610	nd.	positive	91.4%	29.6%	0.7916	2.22E-04
15	cupA fimbriae	PA14_37000	*cupA5*	negative	234.0%	283.0%	0.5970	0.2487
		PA14_37010	*cupA4*		497.8%	253.6%	0.0190	0.0647
		PA14_37030	*cupA3*		131.7%	167.2%	0.6505	0.2187
		PA14_37040	*cupA2*		327.9%	1534.3%	0.1186	3.01E-05
		PA14_37060	*cupA1*		150.4%	209.7%	0.2327	0.0303
		PA14_37070	*crgA*		77.7%	330.8%	0.4439	3.51E-04
		PA14_37080	*crgC*		103.4%	188.5%	0.9086	0.0705
16	unknown	PA14_39480	nd.	positive	39.0%	79.7%	0.0041	0.4959
17	iron transporter	PA14_39800	*femI*	positive	22.6%	9.8%	0.0011	2.42E-09
		PA14_39810	*femR*		87.9%	21.0%	0.7775	7.88E-06
		PA14_39820	*femA*		188.2%	53.0%	0.1775	0.1060
18	T6SS locus 2	PA14_42970	*sfa2*	positive	53.3%	109.1%	0.0574	0.8044
		PA14_42980	*clpV2*		60.1%	95.8%	0.1190	0.9195
		PA14_42990	*hsiH2*		62.1%	75.0%	0.1567	0.4326
		PA14_43000	*hsiG2*		50.2%	92.0%	0.0364	0.8386
		PA14_43020	*hsiF2*		46.4%	85.3%	0.0221	0.7078
		PA14_43030	*hsiC2*		45.8%	99.1%	0.0170	1.0000
		PA14_43040	*hsiB2*		50.3%	104.6%	0.0372	0.8917
		PA14_43050	*hsiA2*		53.6%	53.7%	0.0557	0.0590
		PA14_43070	*hcp2*		83.8%	106.3%	0.6700	0.9442
		PA14_43080	*vgrG2*		98.7%	82.2%	0.2025	1.0000
		PA14_43090	nd.		54.3%	62.1%	0.2342	0.4469
		PA14_43100	nd.		90.1%	122.0%	0.7749	0.5318
19	putative urea transporter	PA14_45060	nd.	negative	1323.4%	97.3%	0.0022	1.0000
20	LasR QS system	PA14_45940	*lasI*	positive	65.1%	81.2%	0.1884	0.5331
		PA14_45950	*rsaL*		57.4%	84.8%	0.0926	0.6313
		PA14_45960	*lasR*		65.8%	82.7%	0.2000	0.5695
21	unknown	PA14_46160	nd.	positive	49.8%	84.7%	0.0348	0.6285
22	putative iron transporter	PA14_46810	nd.	positive	62.0%	85.4%	0.1586	0.6440
		PA14_46820	nd.		65.9%	89.7%	0.2212	0.7592
23	cobalamine biosynthesis	PA14_47650	*cobS*	unclear	113.0%	102.6%	0.7207	0.9564
		PA14_47660	nd.		107.6%	119.6%	0.8194	0.5889
		PA14_47670	*cobT*		97.4%	101.4%	0.9543	0.9522
		PA14_47680	*cobU*		122.6%	95.7%	0.5426	0.9350
		PA14_47690	*cobQ*		124.8%	96.1%	0.4896	0.9324
		PA14_47720	*cobC*		120.7%	97.5%	0.5619	0.9771
		PA14_47730	*cobD*		146.4%	104.9%	0.2585	0.8937
		PA14_47750	nd.		139.4%	125.4%	0.3259	0.5076
		PA14_47760	*cobB*		128.4%	100.3%	0.4330	0.9789
		PA14_47790	*cobO*		137.8%	84.3%	0.3199	0.6177
		PA14_47800	nd.		167.2%	79.0%	0.1103	0.4845
		PA14_47910	nd.		281.2%	149.0%	0.0430	0.3588
		PA14_47920	nd.		71.5%	77.5%	0.7259	0.8343

24	ABC transporter arabinose transport	PA14_47930	nd.	negative	275.5%	101.3%	0.1063	1.0000
		PA14_47940	nd.		93.8%	158.9%	1.0000	0.6625
		PA14_47950	nd.		20.4%	111.6%	0.1569	1.0000
		PA14_47960	nd.		103.1%	115.9%	1.0000	0.7770
		PA14_47970	nd.		67.4%	86.0%	0.3713	0.7021
25	putative antibiotic efflux pump	PA14_48240	nd.	unclear	111.7%	54.7%	0.9045	0.1344
		PA14_48280	nd.		104.6%	99.0%	1.0000	1.0000
		PA14_48300	nd.		305.1%	67.6%	0.0538	0.3947
26	rhamnolipids biosynthesis	PA14_49750	nd.	positive	63.0%	75.3%	0.1847	0.4027
		PA14_49760	*rhlC*		42.9%	74.5%	0.0110	0.3732
27	MvfR QS system	PA14_51340	*mvfR*	positive	66.4%	55.5%	0.2132	0.0725
28	MvfR QS system	PA14_51350	*phnB*	positive	3.9%	12.4%	1.56E-17	2.68E-09
		PA14_51360	*phnA*		2.0%	4.5%	6.44E-23	1.00E-16
29	MvfR QS system	PA14_51380	*pqsE*	positive	1.8%	4.7%	9.56E-24	2.35E-16
		PA14_51390	*pqsD*		2.2%	4.0%	3.91E-22	1.32E-17
		PA14_51410	*pqsC*		1.0%	1.7%	3.29E-28	7.19E-24
		PA14_51420	*pqsB*		1.2%	2.2%	4.33E-26	1.20E-21
		PA14_51430	*pqsA*		0.7%	1.3%	3.46E-30	1.71E-25
30	antioxidant	PA14_51830	*dps*	positive	49.6%	74.3%	0.0316	0.3672
31	phenylalanine catabolism	PA14_52980	nd.	positive	93.0%	103.1%	0.8432	0.9080
		PA14_52990	nd.		50.7%	86.3%	0.0372	0.6624
		PA14_53000	nd.		53.8%	91.6%	0.0588	0.8051
		PA14_53010	nd.		57.0%	90.0%	0.0843	0.7592
32	antioxidant	PA14_53290	*trxB2*	positive	34.8%	21.4%	0.0015	4.68E-06
		PA14_53300	*ahpB*		17.3%	10.3%	5.68E-07	1.37E-10
33	rRNA #2	PA14_55620	*5S rRNA*	negative (see RTqPCR)	na.	na.	na.	na.
		PA14_55631	*23S rRNA*		na.	na.	na.	na.
		PA14_55634	*tRNA-Ala*		na.	na.	na.	na.
		PA14_55635	*tRNA-Ile*		na.	na.	na.	na.
		PA14_55637	*16S rRNA*		na.	na.	na.	na.
34	ABC transporter (arginine?)	PA14_58350	*dppA1*	positive (indirect via blocking PsdR)	77.0%	96.1%	0.4285	0.9206
		PA14_58360	*dppA2*		72.3%	103.2%	0.3290	0.9046
		PA14_58375	*mdpA*		70.1%	63.1%	0.2777	0.1693
		PA14_58380	*psdR*	negative	149.9%	115.3%	0.2382	0.7108
		PA14_58390	*dppA3*	positive (indirect via blocking PsdR)	65.6%	62.8%	0.1949	0.1564
		PA14_58410	*odp*		91.6%	77.8%	0.8012	0.4629
		PA14_58420	*dppA4*		76.7%	109.7%	0.4191	0.7617
		PA14_58440	*dppB*		76.2%	122.0%	0.4088	0.5290
		PA14_58450	*dppC*		78.9%	113.2%	0.4735	0.6960
		PA14_58470	*dppD*		80.1%	119.0%	0.5038	0.5816
		PA14_58490	*dppF*		77.8%	113.1%	0.4476	0.6905
35	rRNA #3	PA14_62050	*5S rRNA*	negative (see RTqPCR)	na.	na.	na.	na.
		PA14_62060	*23S rRNA*		na.	na.	na.	na.
		PA14_62070	*tRNA-Ala*		na.	na.	na.	na.
		PA14_62080	*tRNA-Ile*		na.	na.	na.	na.
		PA14_62090	*16S rRNA*		na.	na.	na.	na.
36	D-alanine metabolism	PA14_70010	nd.	positive	53.9%	105.9%	0.0610	0.8469
		PA14_70040	*dadA*		66.1%	116.9%	0.2056	0.6104
37	rRNA #4	PA14_70870	*5S rRNA*	negative (see RTqPCR)	na.	na.	na.	na.
		PA14_70880	*23S rRNA*		na.	na.	na.	na.
		PA14_70890	*tRNA-Ala*		na.	na.	na.	na.
		PA14_70900	*tRNA-Ile*		na.	na.	na.	na.
		PA14_70910	*16S rRNA*		na.	na.	na.	na.

<u>Supplementary Table S1: RNAseq data showing expression of genes of interest in each of the 37 MvfR binding site.</u>

	primer name	sequence 5' - 3'
for ChIPqPCR	rhlR_ChIP-F	CCCTCATGTGTGTGCTGGTA
	rhlR_ChIP-R	CGATGAAGACTTGATGCCGG
	lasR_ChIP-F	AGTGCGTAGTCCTTGAGCAT
	lasR_ChIP-R	CGTACTGCCGATTTTCTGGG
	phrS_ChIP-F	CTCATGGTCGCTTTCTTCGG
	phrS_ChIP-R	AGGCGATATGTGCAGCCTAT
	hsiA2_ChIP-F	TTGCCCAGCTCATTTTCCAG
	hsiA2_ChIP-R	CATGGTGTGCATTGAAGGGT
	xcpR_ChIP-F	GCTTAAGATAACGGCTCGCC
	xcpR_ChIP-R	AATGGGGCTGTCATCATGGA
	ahpC_ChIP-F	ACTACGGCGAGTTCCAGAAA
	ahpC_ChIP-R	GTTGATCACGAAGGTACCGC
	trxB2_ChIP-F	CCAGGCTCCCGGAAGAATAT
	trxB2_ChIP-R	TCCGACAAGAGCCAGATACC
	dps_ChIP-F	AAGGCCGACTGAAAGGAGAA
	dps_ChIP-R	TGCAGCGTATTGAACATCGG
	infC_ChIP-F	ATTCCGTGCCAAGTCCGA
	infC_ChIP-R	GGCCTTCGATTCAACTTCCC
	rpoD_ChIP-F	ACCGTCGTGGCTACAAATTC
	rpoD_ChIP-R	GGCGATCTTCAGTACCTTGC
for qRT-PCR	rhlR-F1	CTCCTCGGAAATGGTGGTCT
	rhlR-R1	TTCTGGGTCAGCAACTCGAT
	lasR-F1	CGTACTGCCGATTTTCTGGG
	lasR-R1	AGTGCGTAGTCCTTGAGCAT
	phrS-F1	TGTTCATCGACGAAGTGGTT
	phrS-R1	GGCTATGGGAATCCTTCCAG
	hsiA2-F1	GTGTTGAAGCAGGGCATGAA
	hsiA2-R1	AATCCCGATTCGTGCAACTG
	hcp2-F	GGCTTCAACCACGAAGTGAT
	hcp2-R	AGGTGATCTTGCGGTAGGTG
	xcpP-F1	CGGACGACATTACAAGCGAG
	xpcP-R1	TCGCTCGAGTCTTCTTCGG
	xcpR-F1	CAAGCTGATGGACGAAACGG
	xcpR-R1	CCAGGTGGTATTCGATCGGA
	xcpS-F1	GACAGTTCCCGCCGATGAT
	xcpS-R1	GAATGGCAAGGACGATGACC
	16S-F1	GGGAGGAAGGGCAGTAAGTT
	16S-R1	ACCACCCTCTACCGTACTCT
	23S-F1	GGTACTCCGGGGATAACAGG
	23S-R1	CTCTCGTACTAGGAGCAGCC
	5S-F1	GAACCACCTGATCCCTTCCC
	5S-R1	TAGGAGCTTGACGATGACCT
	thrS-F1	CGTGCAGATCTTCAACCAGG
	thrS-R1	TTCTTCACCTGCTCTTCGGT
	infC-F	CATCTCGGCTCGTGAGGTAC
	infC-R	GTAGTCCATGATGCGGCAGA
	rpmI-F1	TGCCAAAGATGAAGACCAAAAGT
	rpmI-R1	CGCAACGTCAGACTTGTTCA

ahpC-F1	ACTACGGCGAGTTCCAGAAA
ahpC-R1	GTTGATCACGAAGGTACCGC
ahpF-F1	GGTCGACATCATGAACCTGC
ahpF-R1	CCTTGGCCTTGTATTCCTGC
ahpB-F1	GATGCCTTCCAGCTTTCCAG
ahpB-R1	GAACTGCGAGTCGATGGAGA
trxB2-F1	TCGGCCATACTCCCAATACC
trxB2-R1	AATCGAGATAGCGCTCCACA
dps-F1	CCGATGTTCAATACGCTGCA
dps-R1	ACGCCTTCCTCTTCCTTGAT
rpoD-F	CTGATCCAGGAAGGCAACAT
rpoD-R	TGAGCTTGTTGATCGTCTCG

Supplementary Table S2: List of primers used for ChIPqPCR or qRT-PCR.
